# Lack of Cardiotoxicity Endpoints in Prospective Trials Involving Chest Radiation Therapy: A Review of Registered, Latter-Phase Studies

**DOI:** 10.3389/fonc.2022.808531

**Published:** 2022-02-09

**Authors:** Rahul N. Prasad, Eric D. Miller, Daniel Addison, Jose G. Bazan

**Affiliations:** ^1^ Department of Radiation Oncology at the Arthur G. James Cancer Hospital and Richard J. Solove Research Institute, The Ohio State University Comprehensive Cancer Center, Columbus, OH, United States; ^2^ Cardio-Oncology Program, Division of Cardiology, The Ohio State University Medical Center, Columbus, OH, United States; ^3^ Division of Cancer Prevention and Control, Department of Internal Medicine, College of Medicine, The Ohio State University, Columbus, OH, United States

**Keywords:** chest radiation therapy, thoracic radiation therapy, cardiotoxicity, clinical trials, major adverse cardiac events, breast cancer, esophageal cancer, lung cancer

## Abstract

**Background:**

Chest radiation therapy (RT) has been associated with increased cardiac morbidity and mortality in numerous studies including the landmark Darby study published in 2013 demonstrating a linear increase in cardiac mortality with increasing mean heart radiation dose. However, the extent to which cardiotoxicity has been incorporated as an endpoint in prospective RT studies remains unknown.

**Methods:**

We queried clincaltrials.gov to identify phase II/III trials in lung, esophageal, lymphoma, mesothelioma, thymoma, or breast cancer from 1/1/2006-2/1/2021 enrolling greater than 100 patients wherein chest RT was delivered in at least one treatment arm. The primary endpoint was the rate of inclusion of cardiotoxicity as a specific primary or secondary endpoint in the pre- (enrollment started prior to 1/1/2014) versus post-Darby era using the Chi-square test (p<0.05 considered significant). We also analyzed clinical trial factors associated with the inclusion of cardiotoxicity as an endpoint using logistic regression analysis.

**Results:**

In total, 1,822 trials were identified, of which 256 merited inclusion. 32% were for esophageal, 31% lung, 28% breast, and 7% lymphoma/thymoma/mesothelioma cancers, respectively. 5% (N=13) included cardiotoxicity as an endpoint: 6 breast cancer, 3 lung cancer, 3 esophageal cancer, and 1 lymphoma study. There was no difference in the inclusion of cardiotoxicity endpoints in the pre-Darby versus post-Darby era (3.9% vs. 5.9%, P=0.46). The greatest absolute increase in inclusion of cardiotoxicity as an endpoint was seen for lung cancer (0% vs. 6%, p=0.17) and breast cancer (5.7% vs. 10.8%, p=0.43) studies, though these increases remained statistically non-significant. We found no clinical trial factors associated with the inclusion of cardiotoxicity as an endpoint.

**Conclusions:**

Among prospective trials involving chest RT, cardiotoxicity remains an uncommon endpoint despite its prevalence as a primary source of toxicity following treatment. In order to better characterize cardiac toxicities, future prospective studies involving chest RT should include cardiotoxicity endpoints.

## Introduction

Cardiovascular disease (CVD) is the leading cause of mortality in cancer survivors (1). Chest radiation therapy (RT) has been associated with increased rates of cardiac morbidity and mortality in survivors of breast (2–4), lung (5, 6), and esophageal cancers (7–9), and lymphoma (10–13) including by the landmark Darby study published in 2013 that demonstrated a linear, persistent increase in cardiac mortality with increasing mean heart radiation dose in patients treated for breast cancer (3). The Darby study was a population-based, case control analysis of 2,168 Scandinavian women who underwent RT for breast cancer from 1958-2001, 963 of whom experienced major coronary events while the remainder without coronary events served as controls (3). This analysis established that excess, major coronary events occurred even within the first 5 years after RT and that these risks persisted for decades.

After publication of this study, increased awareness of this issue motivated numerous other retrospective analyses, including reports in non-small cell lung cancer (NSCLC) demonstrating that a significant proportion of cardiac events occur even within two years of RT completion (5, 6). Additionally, using echocardiography, cohort studies have identified subclinical cardiac dysfunction occurring just months after RT (14, 15). Appreciation of the adverse effects of RT on the heart, largely based on retrospective studies, has led to an increased emphasis on minimizing radiation dose to the heart or its substructures during radiation planning (3, 5, 6, 9, 12, 13, 16). However, the extent to which cardiotoxicity has been incorporated as an endpoint in prospective RT studies remains unknown. We hypothesized that cardiotoxicity has increased in frequency as an endpoint in oncology trials involving thoracic RT since publication of the Darby study in 2013, and we sought to quantify this change in trial design.

## Method

We queried clinicaltrials.gov for all phase II or III interventional studies conducted from 1/1/2006 until 2/1/2021 that included RT for definitive therapy of breast, esophageal, lymphoma, mesothelioma, thymoma, or lung cancer with a planned enrollment of greater than 100 patients. In our query, we specified interventional studies of phase II or III only within the relevant date range and included “radiation” and the type of cancer as additional terms. Smaller, observational, and early-phase studies were excluded. While single arm, observational studies looking at serum and imaging biomarkers provide important hypothesis-generating data, it is difficult to correlate these studies with cardiac-specific outcomes due to the number of patients needed to see any measurable increase in cardiac toxicity. Absolute rates of excess major cardiac events may be low, particularly in certain populations such as patients with breast cancer (4) or lymphoma, and identifying meaningful differences may be beyond the scope of smaller trials. It often takes years for events to occur, which is beyond the funding window of many smaller, early-phase studies that are just looking at early changes in biomarkers. Focusing on phase II/III trials mitigated these issues, and allowed us to assess the rate at which the trials most likely to affect standard of care were considering the potential cardiac consequences of interventions. In contrast, phase IV trials were not considered, because we sought to quantify the rate of inclusion of cardiotoxicity endpoints in the pre-market trials that impact standard of care in oncology. Studies returned by clinicaltrials.gov query were evaluated by an experienced team of 3 researchers with a background in clinical radiation oncology. Studies were stratified into pre-Darby era (enrollment started prior to 1/1/2014) or post-Darby era (enrollment after 1/1/2014). Additional information about each trial was collected, including but not limited to trial phase, type of primary endpoint (cancer control, patient reported toxicity, physician reported toxicity, or other), presence of a cardiotoxicity primary or secondary endpoint, detailed information about RT fractionation and delivery, inclusion of concurrent systemic therapy, current trial recruitment status, country of origin, trial duration, and trial sponsor. We included the report of any cardiac endpoints by trials including both clinical and subclinical outcomes measures.

The primary endpoint of our analysis was the rate of inclusion of any cardiotoxicity endpoint (whether primary or secondary) in trials from the pre-Darby versus post-Darby eras. The Chi-square test was used to compare the rate of inclusion of cardiotoxicity endpoints between groups. We further analyzed whether any clinical trial factors (disease site – breast vs. non-breast; study era – post-Darby vs. pre-Darby; clinical trial phase – III vs. I-II, sample size – dichotomized by the median sample size across all trials; trial duration – dichotomized by the median; and use of concurrent chemotherapy – yes vs. no) were associated with the inclusion of cardiotoxicity as an endpoint with univariate logistic regression analysis. Statistical tests were 2-sided with statistical significance evaluated at the α=0.05 significance level.

## Results

**Figure 1:**
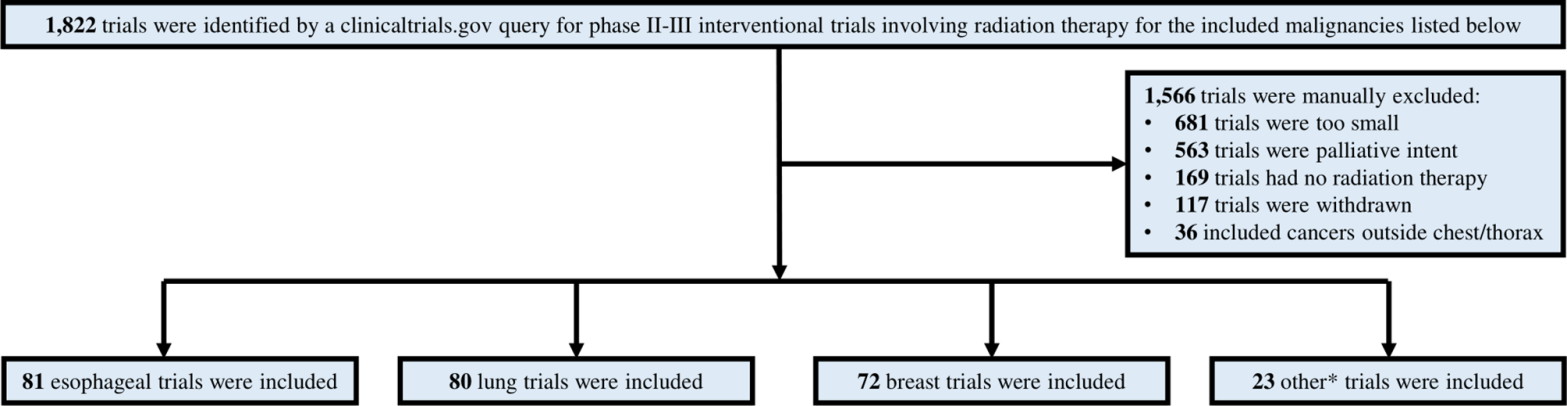
Study schema documenting identification of trials meeting inclusion criteria. *other = lymphoma, mesothelioma, and thymoma.

**Figure 2:**
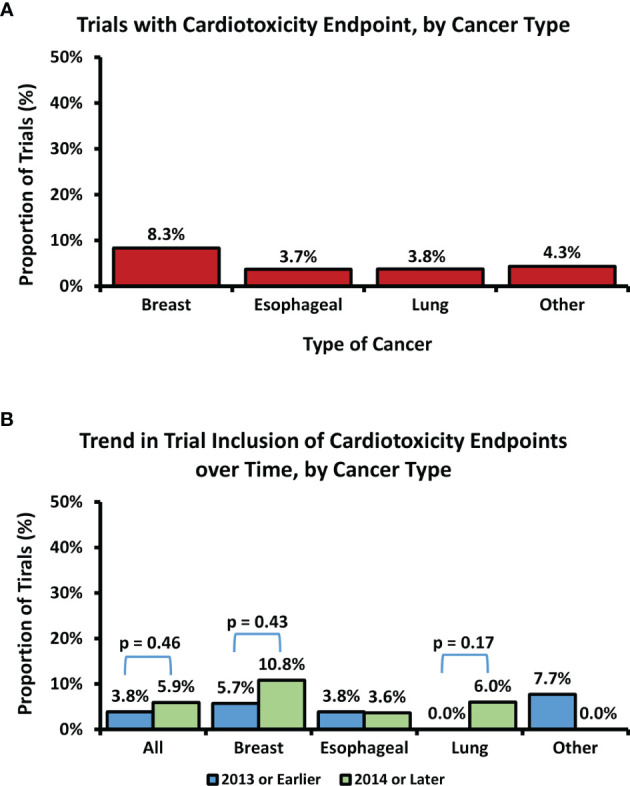
Overall proportion of included trials with a cardiotoxicity endpoint, by cancer type **(A)**. Trend in trial inclusion of cardiotoxicity endpoints over time, by cancer type **(B)**. Differences in rate of inclusion of cardiac endpoints over time are non-significant (p > 0.05).

Overall, 1,822 trials were reviewed, and 256 met the study criteria (**Figure 1**). Of the trials included, 32%, 31%, and 28% involved esophageal, lung, and breast cancers, respectively; detailed characteristics of the included trials, stratified by trial era, are presented in **Table 1**. The remaining 9% were lymphoma, thymoma, or mesothelioma. Across all trials, 59% included concurrent systemic therapy, while 4% and 4% of trials included stereotactic body radiation therapy and proton therapy, respectively. Overall, 5% of included trials (N=13) included cardiotoxicity as an endpoint: 6 breast cancer (8%), 3 lung cancer (4%), 3 esophageal cancer (4%), and 1 lymphoma study (4% of all other included cancers) (**Figure 2A**). The median trial duration was 6.5 years (1.0 - 19.9 years). Of these trials, 5 (2%) included a cardiotoxicity metric as a primary endpoint and 8 (3%) as a secondary endpoint. In general, these endpoints were clinically defined and predominately involved the measurement of serious late effects such as major adverse cardiac events (7 trials, or 54%), including cardiac death and/or ischemic heart disease. A minority of trials evaluated for lower-grade cardiac toxicities. Across all cancer types, there was no statistically significant increase in the inclusion of cardiotoxicity as an endpoint in the pre-Darby versus post-Darby era (3.9% vs. 5.9%, p=0.46). The greatest absolute increases in inclusion of cardiotoxicity as an endpoint were seen for lung cancer (0.0% vs. 5.9%, p=0.17) and breast cancer (5.7% vs. 10.8%, p=0.43) studies, though these increases remained insignificant (**Figure 2B**). Inclusion of cardiotoxicity endpoints in studies of esophageal cancer decreased from 3.8 to 3.6%, and from 7.7% to 0.0% for all other included malignancies. On univariate logistic regression analysis, no variables of interest were associated with increased likelihood of a trial reporting a cardiotoxicity endpoint (**Table 2**). Multivariate analysis was not pursued due to the lack of significant variables on univariate analysis.

**Table 1:** Characteristics of trials meeting inclusion criteria, stratified by era (pre- vs post-Darby publication).

	All Trials	2013 or Earlier	2014 or Later
	N (%)	N (%)	N (%)
**Total**	256 (100)	104 (41)	152 (59)
**Cancer Type**			
Esophagus	81 (32)	26 (25)	55 (36)
Lung	80 (31)	30 (29)	50 (33)
Breast	72 (28)	35 (34)	37 (24)
Other	23 (9)	13 (13)	10 (7)
**Trial Phase**			
II	122 (48)	54 (52)	68 (45)
III	134 (52)	50 (48)	84 (55)
**Primary Endpoint**			
Cancer Control	199 (78)	78 (75)	121 (80)
Patient Reported Toxicity	7 (3)	3 (3)	4 (3)
Physician Reported Toxicity	41 (16)	19 (18)	22 (14)
Other	8 (3)	4 (4)	5 (3)
**Cardiotoxicity Endpoint**			
Primary	5 (2)	1 (1)	4 (3)
Secondary	8 (3)	3 (3)	5 (3)
None	243 (95)	100 (96)	143 (94)
**Concurrent Systemic Therapy**			
Yes	152 (59)	53 (51)	99 (65)
No	104 (41)	51 (49)	53 (35)
**Planned Enrollment**			
100-499	202 (79)	81 (78)	121 (80)
500-999	35 (14)	13 (13)	22 (14)
>1000	18 (7)	9 (9)	9 (6)
**SBRT****^a^** **Included**			
Yes	9 (4)	2 (2)	7 (5)
No	247 (96)	102 (98)	145 (95)
**Proton Therapy Included**			
Yes	10 (4)	7 (7)	3 (2)
No	246 (96)	97 (93)	149 (98)

a Stereotactic body radiation therapy.

**Table 2:** Logistic regression analysis of association of radiation clinical trial characteristics and inclusion of a cardiac primary or secondary endpoint.

Variable	Odds Ratio	95% CI	p-value
**Breast vs. Non-Breast disease site**	2.30	0.75-5.25	0.15
**Phase III vs. Phase II**	2.12	0.64-7.08	0.22
**Use of Concurrent Chemotherapy (Yes vs. No)**	0.41	0.13-1.29	0.13
**Trial Size (>220 patients vs. ≤ 220 patients)**	1.20	0.39-3.66	0.75
**Trial Duration (>6.5 years vs. ≤ 6.5 years)**	0.43	0.13-1.42	0.17
**Post-Darby Era vs. Pre-Darby Era**	1.57	0.47-5.25	0.46

## Discussion

Recent recognition of the adverse cardiac effects of RT has led to an increased focus on minimizing radiation dose to the heart or its substructures (3, 5, 6, 9, 12, 13, 16), yet overall, our analysis found no significant increase in the rate of cardiotoxicity endpoints in clinical trials involving chest RT in the post-Darby era. Even after publication of the Darby study in 2013, roughly 95% of included trials did not include cardiotoxicity endpoints. Examined individually, none of the included malignancies showed a significant change in cardiac endpoint reporting over time. There was a numeric increase in the proportion of trials in breast and lung cancer including cardiotoxicity endpoints, perhaps related to increased awareness from retrospective series and subsequent improved access to funding, but it was ultimately non-significant and overall rates remained objectively low. Surprisingly, studies in the lymphoma/thymoma/mesothelioma showed a numeric decrease in incorporation of cardiotoxicity endpoints over time which most likely relates to the small sample size. These results are startling given the recent trend towards increasing publication of retrospective data detailing the prevalence of cardiac toxicity after chest RT. Of the 250 Pubmed indexed publications identified by a query for “radiation therapy” AND “cardiotoxicity,” over half (128) were published in the last 5 years alone. One possible explanation is that investigators have been prematurely reassured by decreased total doses to the heart seen with modern treatment planning and delivery techniques in breast (17), esophageal (18), and lung (19) cancers. However, even if heart doses are decreasing, it is still important that we adequately monitor cardiac outcomes, so that we can confirm that these lower doses translate to decreased cardiac risk. Ultimately, if cardiac events are adequately measured in the prospective setting and event rates are found to be acceptably low with modern RT techniques, attempts at further decreasing cardiac dose through expensive therapies like proton and heavy ion therapy may be unnecessary. Retrospective studies are useful for hypothesis generation, but due to the inherent biases of such studies, greater prospective characterization of cardiotoxicity after RT is needed.

These findings are highly concerning, because CVD remains the leading cause of non-cancer mortality in cancer survivors (1), and chest RT is consistently linked to increased cardiac complications in survivors of numerous cancers (2–13) perhaps occurring as early as within 2 years of RT completion (5, 6) with elevated risk persisting for decades (2–13). Studies following survivors of various thoracic and chest malignancies suggest that the risk of cardiac complications increases linearly with increasing heart radiation dose (3, 13). Possible complications vary widely depending upon the damaged substructure but include pericarditis (pericardium), heart failure (myocardium), acute coronary syndrome (coronary arteries), valvular disease, and arrhythmia (conduction system) (16). These risks are in addition to known cardiac risks from chemotherapy, and in the modern era, additive risk from concurrent or sequential systemic novel immunotherapies and targeted therapies must also be considered given their increasing links to development of cardiovascular disease (20). The majority of studies included in this analysis included concurrent chemotherapy, immunotherapy, targeted therapy, or hormone therapy.

This analysis has several limitations. This study only evaluated the endpoints from definitive phase II or III trials involving thoracic RT in patients treated with definitive intent with at least 100 patients. The reason for this is that although the Darby et al. study demonstrated a relative increase in at least one acute coronary event of 7.4% per Gray mean heart dose, the absolute increases are quite small especially in patients with no cardiac risk factors. For instance, the Darby et al. study estimates that for a healthy 40-year old woman that receives a mean heart dose of 2 Gy during her breast RT, her risk of at least one acute coronary event by the time she is 80 years old increases by an absolute value of 0.7% (3). Similarly, a follow-up study by Taylor et al. demonstrates that the absolute increase in the risk of death from ischemic heart disease for a healthy 50-year old woman that receives as much as 4 Gy mean heart dose is only 0.3% by the time she is 80-years old (4). This underscores the point that large numbers of patients need to be followed for long periods of time in order to adequately capture the potential effects of thoracic RT on cardiac toxicities. As a result, we did not include single arm prospective studies aimed at identifying serum and/or imaging biomarkers of thoracic RT, because these studies involve small numbers of patients and have limited follow-up to correlate these biomarkers with actual cardiac toxicity. Therefore, not only is it likely that the types of clinical trials that we did not include in our analysis would not have significant rates of inclusion of cardiotoxicity endpoints, but it is also likely that these types of smaller interventional trials would not have adequate power to identify small increases in cardiac toxicities above expected baseline rates. No additional study registries were queried for trials involving chest RT, but in order to best reflect widespread practice, we focused on clinical trials evaluating definitive oncologic therapy as captured by the US-based clinicaltrials.gov. Additionally, study protocols could have been reviewed to determine the rate at which trials without cardiac endpoints were still monitoring for adverse cardiac events. However, such an approach has limited utility, as prior work suggests that cardiotoxicity is underreported by clinical trials that are not specifically designed to characterize cardiac events (21–23). Dichotomizing the comparison eras differently may have impacted the significance of the trend in inclusion of endpoints over time but would not affect the overarching conclusion that clinical trials including cardiac outcomes are too rare.

In summary, among prospective clinical trials involving chest RT, cardiotoxicity remains an uncommon endpoint despite its prevalence as a primary source of toxicity following treatment. While inclusion of cardiotoxicity endpoints has increased slightly over time (numeric increase not achieving significance), overall rates of inclusion among latter-phase trials remain objectively low. Thus, in order to better characterize cardiac toxicities, education is needed to increase researchers’ and clinicians’ awareness of this subject. Additionally, future prospective studies involving chest RT should include cardiotoxicity endpoints with greater frequency.

## Data Availability Statement

The raw data supporting the conclusions of this article will be made available by the authors, without undue reservation.

## Author Contributions

RP and EM: first authors. DA: contributor. JB: senior authorship. All authors contributed to the article and approved the submitted version.

## Funding

This work was supported in part by National Cancer Institute grant P30 CA016058. Dr. Addison is supported by the following grants from the National Institutes of Health: K12CA133250 and K23-HL155890. Dr. Addison is also supported by an American Heart Association–Robert Wood Johnson Foundation Faculty Development Program grant.

## Conflict of Interest

The authors declare that the research was conducted in the absence of any commercial or financial relationships that could be construed as a potential conflict of interest.

## Publisher’s Note

All claims expressed in this article are solely those of the authors and do not necessarily represent those of their affiliated organizations, or those of the publisher, the editors and the reviewers. Any product that may be evaluated in this article, or claim that may be made by its manufacturer, is not guaranteed or endorsed by the publisher.
